# Therapeutic hypothermia versus normothermia in adult patients with traumatic brain injury: a meta-analysis

**DOI:** 10.1186/s40064-016-2391-2

**Published:** 2016-06-21

**Authors:** Youfeng Zhu, Haiyan Yin, Rui Zhang, Xiaoling Ye, Jianrui Wei

**Affiliations:** Department of Intensive Care Unit, Guangzhou Red Cross Hospital, Medical College, Jinan University, Guangzhou, 510220 Guangdong China; Department of Cardiology, Guangzhou Red Cross Hospital, Medical College, Jinan University, Tongfuzhong Road No. 396, Guangzhou, 510220 Guangdong China

**Keywords:** Therapeutic hypothermia, Traumatic brain injury, Mortality, Adult, Meta-analysis

## Abstract

**Introduction:**

Many single-center studies and meta-analyses demonstrate that therapeutic hypothermia (TH), in which the body temperature is maintained at 32–35°C, exerts significant neuroprotection and attenuates secondary intracranial hypertension after traumatic brain injury (TBI). In 2015, two well-designed multi-center, randomized controlled trials were published that did not show favorable outcomes with the use of TH in adult patients with TBI compared to normothermia treatment (NT). Therefore, we performed an updated meta-analysis to assess the effect of TH in adult patients with TBI.

**Methods:**

We reviewed the PubMed, EMbase, Cochrane Central Register of Controlled Trials, China National Knowledge Infrastructure, and Wanfang Databases. We included randomized controlled trials that compared TH and NT in adult patients with TBI. Two reviewers assessed the quality of each study and independently collected the data. We performed the meta-analysis using the Cochrane Collaboration’s RevMan 5.3 software.

**Results:**

We included 18 trials involving 2177 patients with TBI. There was no significant heterogeneity among the studies. TH could not decrease mortality at 3 months post-TBI (RR 0.95; 95 % CI 0.59, 1.55; z = 0.19, P = 0.85) or 6 months post-TBI (RR 0.96; 95 % CI 0.76, 1.23; z = 0.29, P = 0.77). There were no significant differences in unfavorable clinical outcomes when TH was compared to NT at 3 months post-TBI (RR 0.79; 95 % CI 0.56, 1.12; z = 1.31, P = 0.19) or 6 months post-TBI (RR 0.80; 95 % CI 0.63, 1.00; z = 1.92, P = 0.05). TH was associated with a significant increase in pneumonia (RR 1.51; 95 % CI 1.12, 2.03; z = 2.72, P = 0.006) and cardiovascular complications (RR 1.75; 95% CI 1.14, 2.70; z = 2.54, P = 0.01).

**Conclusions:**

Therapeutic hypothermia failed to demonstrate a decrease in mortality and unfavorable clinical outcomes at 3 or 6 months post-TBI. Additionally, TH might increase the risk of developing pneumonia and cardiovascular complications.

## Background

Traumatic brain injury (TBI) is a major cause of death and disability in the younger population and is a great economic and social burden in modern society. Recent studies showed a 21 % increase in the incidence of TBI during the past five years (Andrews et al. 2015). However, effective strategies are few for early care of this disease. Secondary elevations in intracranial pressure (ICP) are frequent in patients with severe TBI and can cause poor outcomes. Thus, the Brain Trauma Foundation (BTF) guidelines from 2007 suggest maintaining an ICP below 20–25 mmHg (Brain Trauma Foundation et al. 2007).

Therapeutic hypothermia (TH), also termed target temperature management (TTM), is the controlled lowering of core body temperature to below 36 °C and is currently recommended by many guidelines for hypoxic ischemic encephalopathy and cardiac arrest (Michael 2013; Crossley et al. 2014). Many animal and single-center studies have demonstrated that therapeutic hypothermia, in which the body temperature is maintained at 32–35°C, exerts significant neuroprotection and attenuates secondary intracranial hypertension after TBI (Soukup et al. 2002; Oddo et al. 2009; Colbourne et al. 2003; Dietrich and Bramlett 2010; Truettner et al. 2011). The effects of TH may include a reduction in cerebral metabolic rate of oxygen (Soukup et al. 2002) and cerebral glucose demand (Soukup et al. 2002; Colbourne et al. 2003), a reduction in calcium influx into the brain cells and the release of excitotoxic amino acids (Dietrich and Bramlett 2010), and the inhibition of early molecular cascades and the stress response, thus preventing apoptosis (Truettner et al. 2011).

Two recent meta-analyses published in 2014 (Crossley et al. 2014; Li and Yang 2014) showed that TH might be effective in reducing death and unfavorable clinical outcomes. However, there were also many controversies. Conflicting results and several negative randomized controlled trials (Shiozaki et al. 1993; Clifton et al. 1993; Marion et al. 1997; Jiang et al. 2000; Clifton et al. 2001; Shiozaki et al. 2001; Yan and Tang 2001; Clifton et al. 2011) have occurred. Moreover, concerns about the potential increased risk of pneumonia following the induction of TH are evident (Sydenham et al. 2009; Woo et al. 2014)^.^

In 2015, two well designed multi-center, randomized controlled trials were published (the Brain-Hypothermia Study, BHYPO trial (Maekawa et al. 2015); the European Study of Therapeutic Hypothermia for Intracranial Pressure Reduction after Traumatic Brain Injury, the Eurotherm3235Trial (Andrews et al. 2015)) that did not show favorable outcomes with the use of TH in patients with TBI.

In addition, a recent prospective study (Mtaweh et al. 2014) indicated that the energy metabolism rate of children is lower than that of adults, which might make the feasibility and efficacy of TH different for adult patients. Therefore, in the present meta-analysis, we aimed to reassess the effect of TH on mortality, unfavorable clinical outcomes (defined as death, a persistent vegetative state, or severe disability) and complications in adult patients with TBI compared to normothermia treatment (NT).

## Methods

### Data sources and search strategy

We reviewed studies published in the Pubmed, EMbase, Cochrane Central Register of Controlled Trials, China National Knowledge Infrastructure and the Wanfang databases. To avoid missing trials, we also searched the references from relevant articles. The keywords and MeSH and Emtree terms used in different combinations for the searches, with limitations set to randomized controlled trials, were “hypothermia”, “target temperature management”, “moderate hypothermia”, “moderate temperature”, “adult”, “traumatic brain injur*”, “head injur*”, “brain injuries”[MeSH]; “traumatic brain injury”[Emtree]; “moderate hypothermia, induced”[MeSH]; and “hypothermia”[MeSH/Emtree]. No limits for language, sample size, gender or the location of the original study were entered for the search.

### Study selection

We determined the publications that were suitable for the meta-analysis using selection criteria as follows: (1) Randomized controlled trial (RCT); (2) Population: hospitalized adult patients with TBI (as in a previous study (Crossley et al. 2014), we defined adult as being the legal age for consent in the country where the trial was conducted); (3) Comparison between therapeutic hypothermia (32–35°C) and normothermia; and (4) Evaluation of mortality or unfavorable clinical outcomes at 3 or 6 months post-TBI. Unfavorable clinical outcomes included death, persistent vegetative state or severe disability that was classified by the Glasgow Outcome Scale. Additionally, variables were compared as follows: incidence of new pneumonia, cardiovascular complications and bleeding complications. All analyses were based on previously published studies; thus, ethical approval and patient consent were not required.

### Data extraction and quality assessment

Two independent reviewers (Rui Zhang and Haiyan Yin) screened the titles and abstracts using a structured data abstraction form, which resulted in high and satisfactory inter-observer agreement. Any disagreement was resolved by consensus or by consulting a third author (Jianrui Wei). We extracted the authors’ names, title of the article, journal in which the study was published, country and year of the study, methodological variables and clinical outcomes. The modified Jadad score was used to evaluate the quality of the included trials. Two independent reviewers (Youfeng Zhu and Xiaoling Ye) assessed the bias of the included studies according to the methods described in the Cochrane Handbook for Systematic Reviews of Interventions (Marion et al. 1993). The parameters were assessed as follows: random sequence generation, blinding of participants and personnel, allocation concealment, blinding of outcome assessment, incomplete outcome data and selective outcome reporting. According to the Cochrane Handbook, other sources of bias were related to the specific trial design used or the early termination of the study due to an extreme baseline imbalance in the selected patients. Because of the nature of these trials, the blind method could not be implemented.

### Statistical analysis

The Cochrane Collaboration’s Review Manager Software 5.3 (RevMan 5.3) was used for the meta-analysis. The results were obtained by direct extraction or by indirect calculation. The risk ratios (RR) and 95 % confidence intervals (CI) were calculated for the binary data, and the standardized mean differences (SMD) and 95% CI were calculated for the continuous data variables. Heterogeneity between trials was tested using the Chi square test, with P < 0.05 and *I*^2^ greater than 50 % indicating significant heterogeneity (Mtaweh et al. 2014). The random effects model was used to calculate the outcomes of both the binary and continuous variables, regardless of statistical heterogeneity. Forest plots were used to graphically display the results. A funnel plot was used to uncover potential publication bias.

## Results

**Fig. 1:**
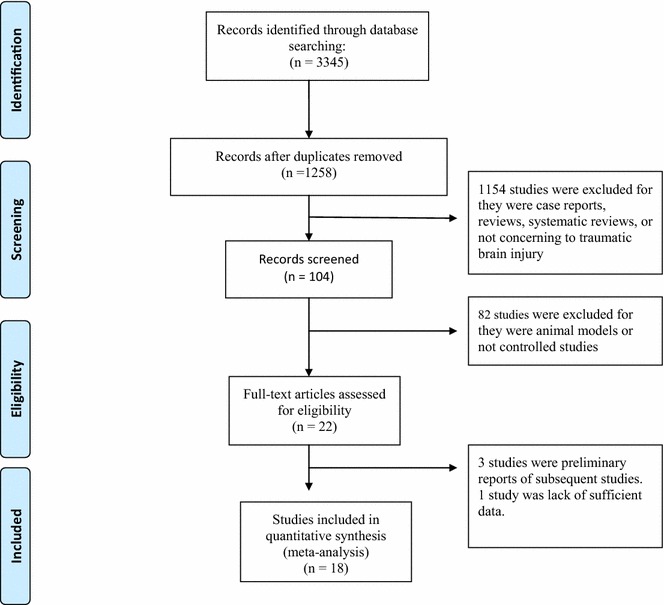
PRISMA flow diagram of the study selection process

Figure 1 shows the selection process for the eligible trials. Initially, 3345 records were identified. After removing duplicate records, animal studies, case reports, review articles, comments, or studies that were not randomized controlled trials, 22 studies remained for assessment. Three studies were preliminary reports of subsequent studies (Liu et al. 2006; Flynn et al. 2015; Marion et al. 1993) and were excluded to avoid duplication. One study did not report the length of the follow-up period and incidence of complications, and was excluded (Yan and Tang 2001). Finally, 18 studies were included in the present meta-analysis (Andrews et al. 2015; Shiozaki et al. 1993; Clifton et al. 1993; Marion et al. 1997; Jiang et al. 2000; Clifton et al. 2001; Shiozaki et al. 2001; Clifton et al. 2011; Maekawa et al. 2015; Gal et al. 2002; Zhi et al. 2003; Qiu et al. 2005, 2006, 2007; Hashiguchi et al. 2003; Lee et al. 2010; Shiozaki et al. 1999; Zhao et al. 2011). The qualities of the included RCTs are shown in Table 1.

**Table 1:** Quality of included studies

References	Randomization method	Blind method	Allocation concealment	Withdrawals/dropouts (NG/NJ)	Jadad score
Clifton et al. (1993)	Method not mentioned	Not used	Method not mentioned	Yes	3
Shiozaki et al. (1993)	Method not mentioned	Not used	Method not mentioned	Yes	3
Marion et al. (1997)	Block randomization	Not used	Method not mentioned	Yes	4
Shiozaki et al. (1999)	Method not mentioned	Not used	Method not mentioned	Yes	3
Jiang et al. (2000)	Method not mentioned	Not used	Method not mentioned	Yes	3
Shiozaki et al. (2001)	Method not mentioned	Not used	Method not mentioned	Yes	3
Clifton et al. (2001)	Method not mentioned	Not used	Method not mentioned	Yes	3
Gal et al. (2002)	Method not mentioned	Not used	Method not mentioned	Yes	3
Zhi et al. (2003)	Method not mentioned	Not used	Method not mentioned	Yes	3
Hashiguchi et al. (2003)	Method not mentioned	Not used	Method not mentioned	Yes	3
Qiu et al. (2005)	Method not mentioned	Not used	Method not mentioned	Yes	3
Qiu et al. (2006)	Method not mentioned	Not used	Method not mentioned	Yes	3
Qiu et al. (2007)	Randomization table	Not used	Yes	Yes	5
Lee et al. (2010)	Method not mentioned	Not used	Method not mentioned	Yes	3
Zhao et al. (2011)	Method not mentioned	Not used	Method not mentioned	Yes	3
Clifton et al. (2011)	Random number generator	Not used	Yes	Yes	5
Andrews et al. (2015)	A central internet or telephone based randomization service	Not used	Yes	Yes	5
Maekawa et al. (2015)	Computer-generated Randomization number	Not used	Method not mentioned	Yes	4

The modified Jadad score was used to evaluate the quality of included trials

A total of 2177 patients with TBIs were included in the present meta-analysis. Of these cases, 1122 patients were randomly assigned to a TH group, and 1055 patients were assigned to an NT group. The characteristics and basic demographic parameters of all patients are shown in Table 2.

**Table 2:** Characteristics and demographic parameters of patients in the included studies

References	N	Age (years)	Gender (M/F)	Target T (°C)	Time from TBI to strating TH (h)	Duration of hypothermia (h)	Duration of rewarming
Clifton et al. (1993)							
Hypothermia	24	16–55	Unknown	32–33	6	48	1 °C/4 h
Normothermia	22	16–60	Unknown				
Shiozaki et al. (1993)							
Hypothermia	16	35.3 ± 15.3	6 M, 10 F	34	5–6	>48	>24 h
Normothermia	17	35.4 ± 12.6	10 M, 7 F				
Marion et al. (1997)							
Hypothermia	40	31 ± 12	36 M, 4 F	32–33	Unknown	24	<1 °C/h
Normothermia	42	35 ± 15	33 M, 9 F				
Shiozaki et al. (1999)							
Hypothermia	8	31.4 ± 12.7	8 M, 0 F	33.5–34.5 (intracranial)	Unknown	48	1 °C/day
Normothermia	8	40.3 ± 23.1	5 M, 3 F				
Jiang et al. (2000)							
Hypothermia	43	42.2	35 M, 8 F	33–35	15	72–366	1 °C/h
Normothermia	44	40.6	37 M, 7 F				
Clifton et al. (2001)							
Hypothermia	199	31 ± 12	Unknown	33	6	48	0.25 °C/h
Normothermia	193	32 ± 13	Unknown				
Shiozaki et al. (2001)							
Hypothermia	45	35 ± 20	35 M, 10 F	34	Unknown	48	1 °C/24 h
Normothermia	46	32 ± 17	31 M, 15 F				
Gal et al. (2002)							
Hypothermia	15	Unknown	Unknown	34	15	72	Unknown
Normothermia	15	Unknown	Unknown				
Hashiguchi et al. (2003)							
Hypothermia	9	29 ± 14.9	9 M, 0 F	34 (intracranial)	Unknown	48	1 °C/day
Normothermia	8	39.1 ± 13.2	5 M, 3 F				
Zhi et al. (2003)							
Hypothermia	198	43 ± 17	Unknown	32–35	9	62.4	0.25 °C/h
Normothermia	198	42 ± 19	Unknown				
Qiu et al. (2005)							
Hypothermia	43	40	26 M, 17 F	33–35	Unknown	72–120	Unknown
Normothermia	43	42.3	30 M, 13 F				
Qiu et al. (2006)							
Hypothermia	45	40.1 ± 9.8	29 M, 20 F	33–35	Unknown	72	8–20 h
Normothermia	45	41.8 ± 11.7	30 M, 15 F				
Qiu et al. (2007)							
Hypothermia	40	41.3	25 M, 15 F	33–35	4.1	96	10–24 h
Normothermia	40	40.2	27 M, 13 F				
Lee et al. (2010)							
Hypothermia	29	44.0 ± 15.1	17 M, 12F	33–35	Unknown	Unknown	Unknown
Normothermia	16	43.5 ± 16.4	10 M, 6 F				
Zhao et al. (2011)							
Hypothermia	40	36.9 ± 14.8	29 M, 10 F	32.7	Within 24	72	Unknown
Normothermia	41	37.5 ± 15.2	30 M, 11 F				
Clifton et al. (2011)							
Hypothermia	52	26	Unknown	33–35	1.6	48	0.5 °C/2 h
Normothermia	45	31	Unknown				
Maekawa et al. (2015)							
Hypothermia	98	39 ± 19	69 M, 29 F	32–34	Within 2	≥72	1 °C/1 day
Normothermia	50	39 ± 18	34 M, 16 F				
Andrews et al. (2015)							
Hypothermia	191	37.4 ± 15.4	Unknown	32–35	Unknown	>48	1 °C/4 h
Normothermia	189	36.7 ± 14.9	Unknown				

Plus–minus values are mean ± SD

*M* male, *F* female, *T* temperature, *N* number, *TH* therapeutic hypothermia

**Fig. 2:**
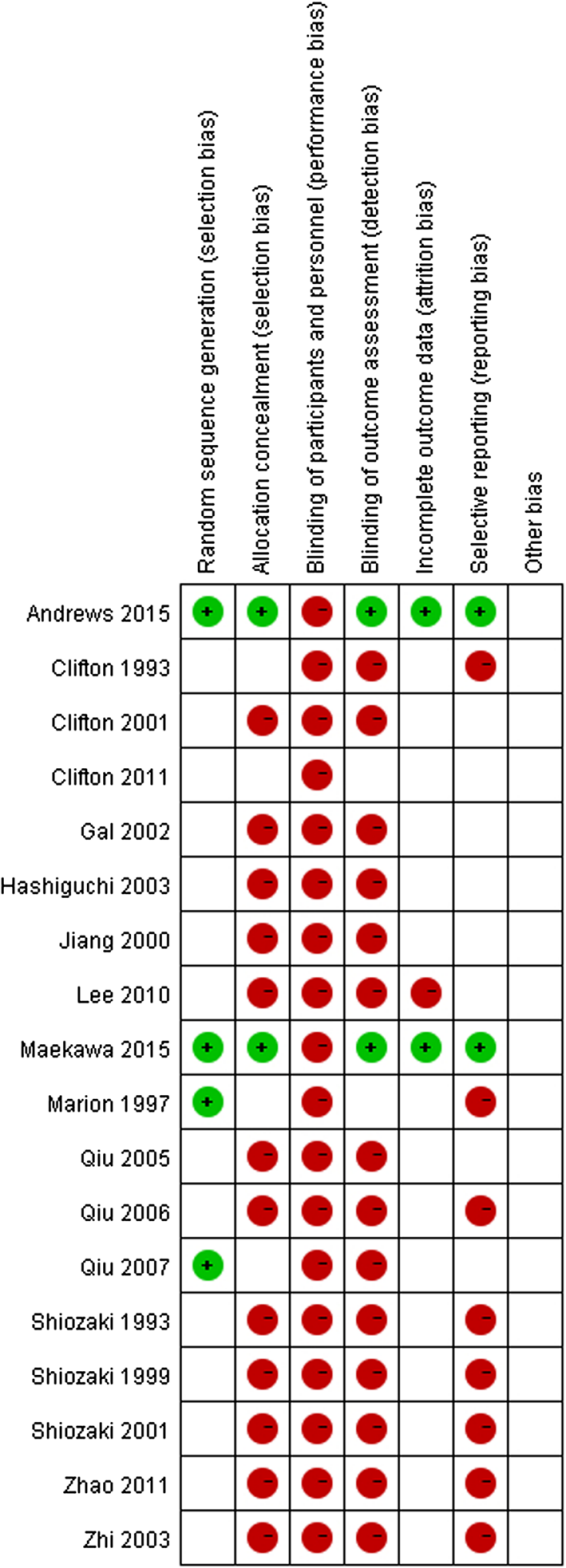
Risk of bias summary: authors’ judgments of the risk of bias for each item in each included study

*Risk of bias in the included studies* We used a tool from the Cochrane Collaboration to assess the risk of bias for each study and presented the details of the results in Fig. 2.

## Effects of mortality

All but one of the included studies (Gal et al. 2002) reported the mortality at the end of the follow-up period. Due to variations in trial protocol, the length of the long-term follow-up period usually varied between 3 and 6 months. Of these included studies, 4 studies reported mortality at 3 months after TBI, 10 studies reported mortality at 6 months after TBI, 3 studies reported mortality at 1 year after TBI, and 1 study reported mortality at 2 years after TBI; the length of the follow-up period was unclear in 1 studies (Lee et al. 2010). We analysed mortality at 3 and 6 months post-TBI.

### Mortality at 3 months post-TBI

**Fig. 3:**
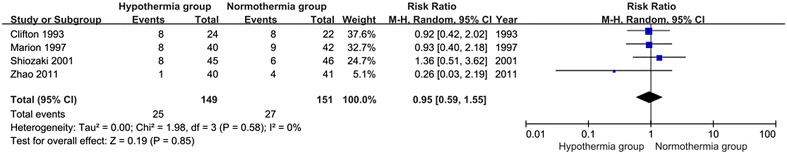
Mortality at 3 months post-TBI between the TH and NT groups

For the analysis of mortality at 3 months post-TBI, 4 trials involving 300 patients were included. When the results of the 4 studies were statistically aggregated, no significant heterogeneity was observed (Chi^2^ = 1.98, df = 3, P = 0.58; *I*^2^ = 0 %) among the studies and therapeutic hypothermia was not associated with a significant reduction in mortality (RR 0.95; 95 % CI 0.59, 1.55; z = 0.19, P = 0.85, Fig. 3).

### Mortality at 6 months post-TBI

**Fig. 4:**
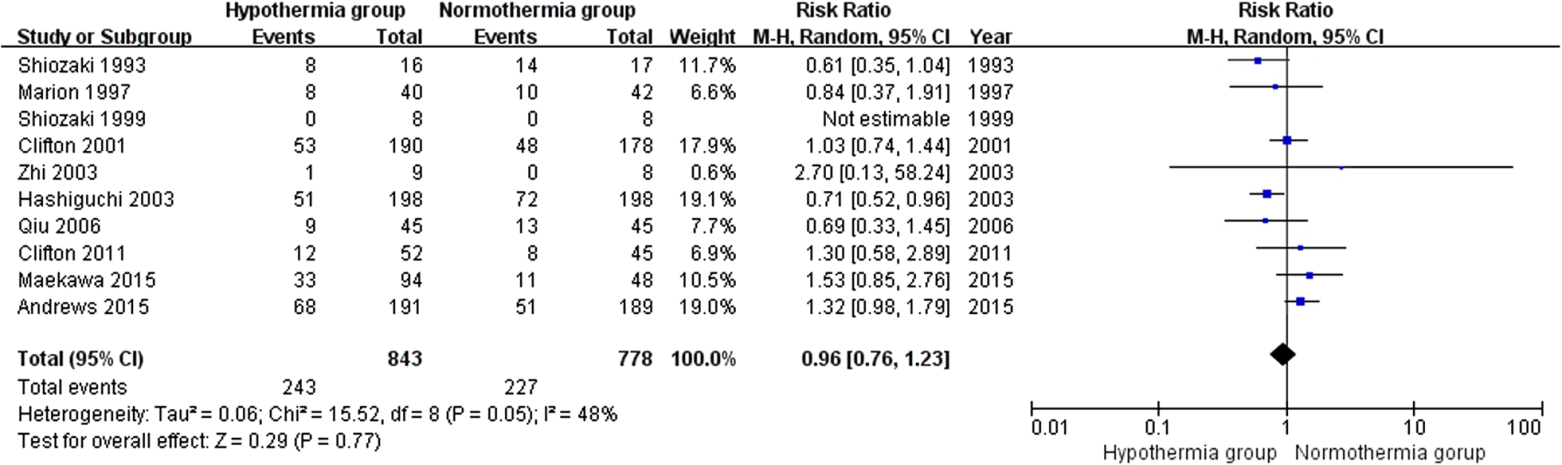
Mortality at 6 months post-TBI between the TH and NT groups

For the analysis of mortality at 6 months post-TBI, 10 trials involving 1621 patients were included. When the results of the 10 studies were statistically aggregated, no significant heterogeneity was observed (Chi^2^ = 15.52, df = 8, P = 0.05; *I*^2^ = 48 %) among the studies and therapeutic hypothermia was not associated with a significant reduction in mortality (RR 0.96; 95 % CI 0.76, 1.23; z = 0.29, P = 0.77, Fig. 4).

### Mortality in trials with a lower risk of bias

**Fig. 5:**
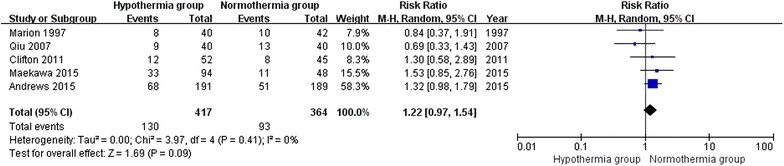
Mortality in trials with a lower risk of bias between the TH and NT groups

For mortality at the final follow-up in trials assessed as having a lower risk of bias (modified Jadad score >3), 5 trials involving 781 patients were included in this sub-analysis. When the results of the 5 studies were statistically aggregated, no significant heterogeneity was observed (Chi^2^ = 3.97, df = 4, P = 0.41; *I*^2^ = 0 %) among the studies and therapeutic hypothermia was not associated with a significant reduction in mortality (RR 1.22; 95 % CI 0.97, 1.54; z = 1.69, P = 0.09, Fig. 5).

## Effects of unfavorable clinical outcomes

All of the included studies reported unfavorable clinical outcomes at the end of the follow-up period. Due to variations in trial protocol, the length of the long-term follow-up period usually varied between 3 and 6 months. Of these included studies, 4 studies reported unfavorable clinical outcomes at 3 months after TBI, 11 studies reported unfavorable clinical outcomes at 6 months after TBI, 2 studies reported unfavorable clinical outcomes at 1 year after TBI, and 1 study reported unfavorable clinical outcomes at 2 years after TBI; the length of the follow-up period was unclear in 1 study (Lee et al. 2010). We analysed unfavorable clinical outcomes at 3 and 6 months post-TBI.

### Unfavorable clinical outcomes at 3 months post-TBI

**Fig. 6:**
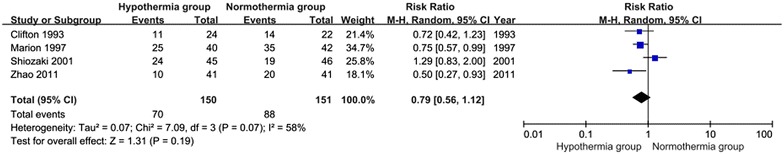
Unfavorable clinical outcomes at 3 months post-TBI between the TH and NT groups

For the analysis of unfavorable clinical outcomes at 3 months after TBI, 4 trials involving 300 patients were included. When the results of the 4 studies were statistically aggregated, significant heterogeneity was observed (Chi^2^ = 7.09, df = 3, P = 0.07; *I*^2^ = 58 %) among the studies and no significant difference between the TH and NT groups was observed (RR 0.79; 95 % CI 0.56, 1.12; z = 1.31, P = 0.19, Fig. 6).

### Unfavorable clinical outcomes at 6 months post-TBI

**Fig. 7:**
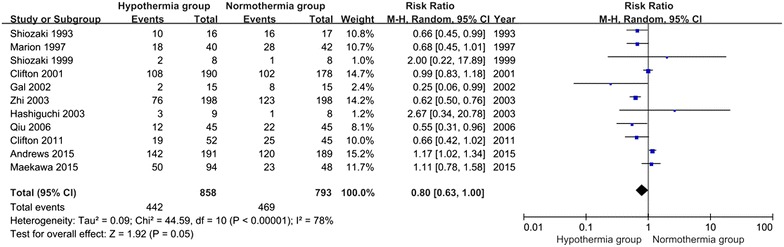
Unfavorable clinical outcomes at 6 months post-TBI between the TH and NT groups

For the analysis of unfavorable clinical outcomes at 6 months post-TBI, 11 trials involving 1651 patients were included. When the results of the 11 studies were statistically aggregated, significant heterogeneity was observed (Chi^2^ = 44.59, df = 10, P < 0.001; *I*^2^ = 78 %) among the studies and no significant difference between the TH and NT groups was observed (RR 0.80; 95 % CI 0.63, 1.00; z = 1.92, P = 0.05, Fig. 7).

### Unfavorable clinical outcomes in trials with a lower risk of bias

**Fig. 8:**
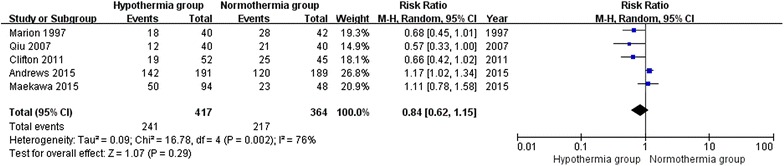
Unfavorable clinical outcomes in trials with a lower risk of bias between the TH and NT groups

For the analysis of unfavorable clinical outcomes at the final follow-up in trials assessed as a lower risk of bias (modified Jadad score >3), 5 trials involving 781 patients were included in this sub-analysis. When the results of the 5 studies were statistically aggregated, significant heterogeneity was observed (Chi^2^ = 16.78, df = 4, P = 0.002; *I*^2^ = 76 %) among the studies and no significant difference was observed between the TH and NT groups (RR 0.84; 95 % CI 0.62, 1.15; z = 1.07, P = 0.29, Fig. 8).

## Pneumonia complications

**Fig. 9:**
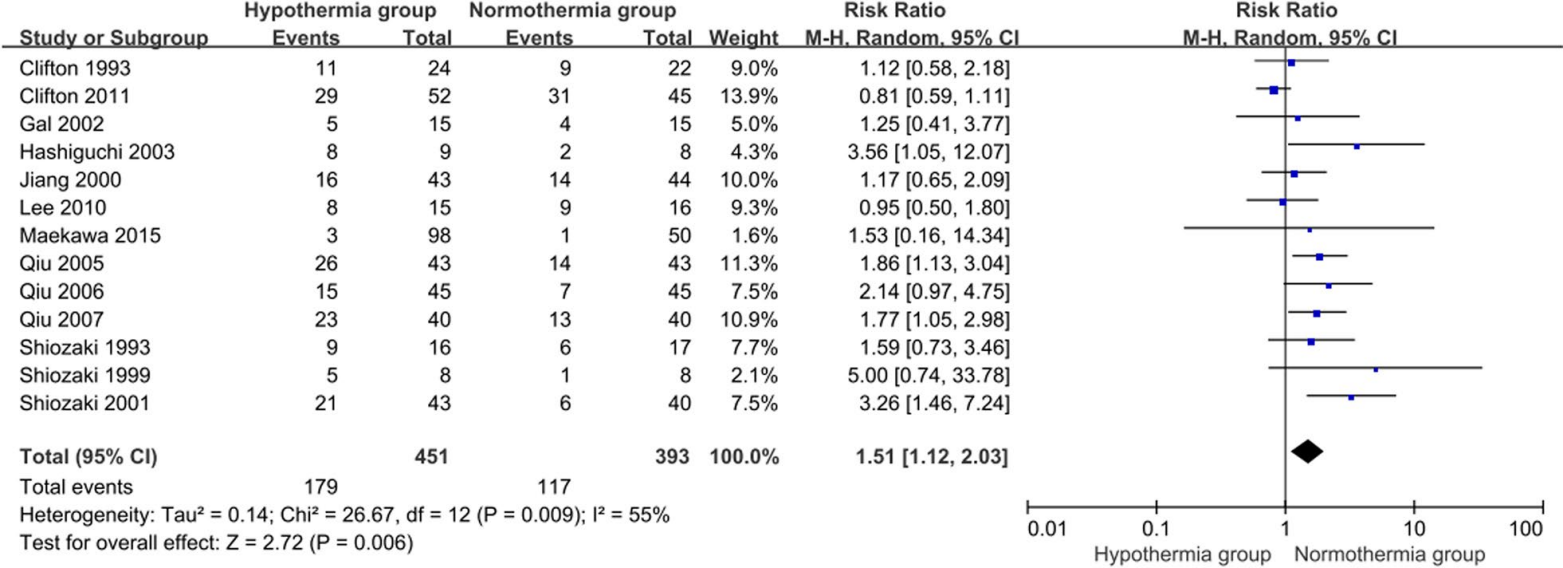
Pneumonia complications between the TH and NT groups

A total of 13 RCTs were included involving 844 patients who reported pneumonia complications. Significant heterogeneity was observed (Chi^2^ = 26.67, df = 12, P = 0.009; *I*^2^ = 55 %) among the 13 trials. In the random effects model, the TH group was associated with a higher risk of developing pneumonia than the NT group (RR 1.51; 95 % CI 1.12, 2.03; z = 2.72, P = 0.006, Fig. 9).

## Cardiovascular complications

**Fig. 10:**
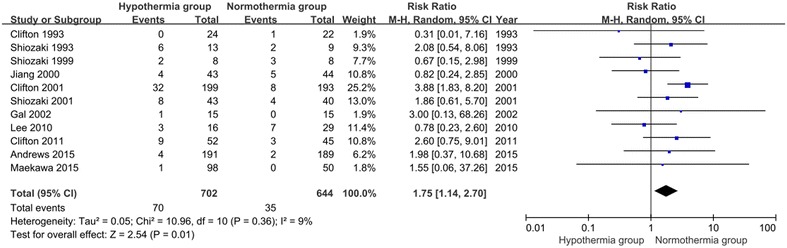
Cardiovascular complications between the TH and NT groups

A total of 11 included studies involving 1346 patients reported cardiovascular complications. No significant heterogeneity was observed (Chi^2^ = 10.96, df = 10, P = 0.36; *I*^2^ = 9 %) among the 11 trials. In the random effects model, the TH group was associated with a higher risk of developing cardiovascular complications than the NT group (RR 1.75; 95 % CI 1.14, 2.70; z = 2.54, P = 0.01, Fig. 10).

## Bleeding complications

**Fig. 11:**
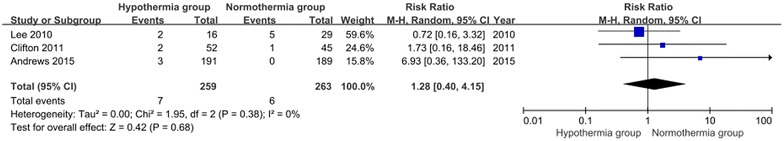
Bleeding complications between the TH and NT groups

A total of 3 RCTs were included involving 522 patients who reported bleeding complications. No significant heterogeneity was observed (Chi^2^ = 1.95, df = 2, P = 0.38; *I*^2^ = 0 %) among the 3 trials. In the random effects model, no significant difference in bleeding complications between the TH and NT groups was observed (RR 1.28; 95 % CI 0.40, 4.15; z = 0.42, P = 0.68, Fig. 11).

**Fig. 12:**
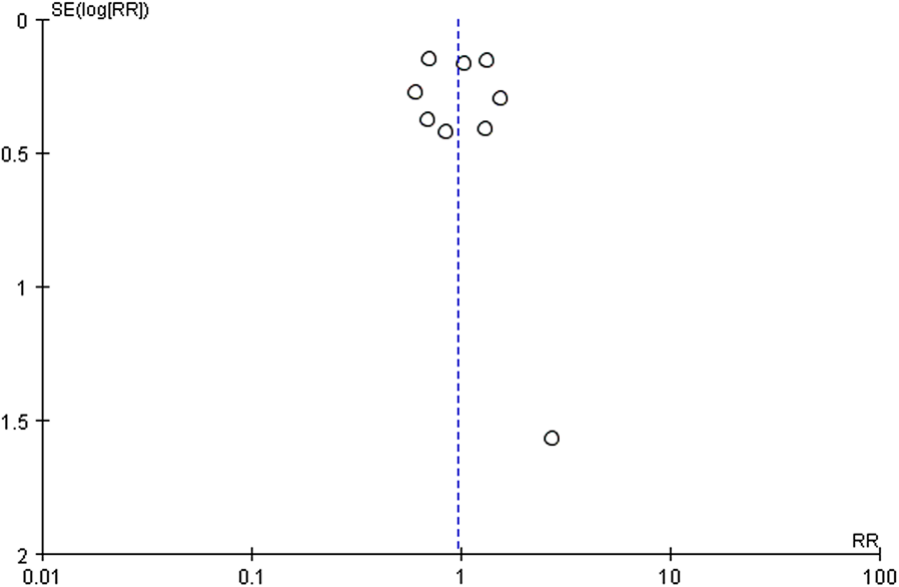
Funnel plot for publication bias

No publication bias was observed based on a visual inspection of the funnel plot (Fig. 12).

## Discussion

Many previous studies and meta-analyses (Crossley et al. 2014; Li and Yang 2014) have assessed the effect of TH compared to NT in TBI patients, and there were contradictory results. This meta-analysis involved 18 studies including 2177 adult patients with TBI (1122 in the TH group and 1055 in the NT group) to further investigate the effectiveness of TH for the treatment of TBI.

The two meta-analyses published in 2014 (Crossley et al. 2014; Li and Yang 2014) showed that TH might be effective in the treatment of TBI, could decrease mortality and could be associated with a reduction in unfavorable clinical outcomes compared to NT. No significant increases were observed in the development of pneumonia complications in TH compared to NT (Crossley et al. 2014). The results of present study were different from previous studies (Crossley et al. 2014; Li and Yang 2014).

The present meta-analysis indicated that TH could not decrease the mortality at 3 months post-TBI (RR 0.95; 95 % CI 0.59, 1.55; z = 0.19, P = 0.85) or the mortality at 6 months post-TBI (RR 0.96; 95 % CI 0.76, 1.23; z = 0.29, P = 0.77) in adult patients with TBI. Additionally, There were no significant differences in unfavorable clinical outcomes at 3 months post-TBI (RR 0.79; 95 % CI 0.56, 1.12; z = 1.31, P = 0.19) or 6 months post-TBI (RR 0.80; 95 % CI 0.63, 1.00; z = 1.92, P = 0.05) when TH was compared to NT. Furthermore, TH was associated with a significant increase in pneumonia complications (RR 1.51; 95 % CI 1.12, 2.03; z = 2.72, P = 0.006) and cardiovascular complications (RR 1.75; 95 % CI 1.14, 2.70; z = 2.54, P = 0.01). The findings suggesting possible harm of hypothermia could be due to a biologic effect of hypothermia or due to the harms or benefits of the other therapies used differentially in the two groups (Andrews et al. 2015). The results of the present study might lead to further understanding of TH in adult patients with TBI and should be interpreted with great caution.

Furthermore, there are still many debates regarding the recent two RCTs. The BHYPO trial was stopped early before the scheduled sample size (300 cases) was reached because of a concern about a shortage of TBI patients (95 cases). The actual sample size was far below the intended target, which might produce bias. For the Eurotherm3235 trial, there were many more debates. Kiwon Lee considered that hypothermia might be helpful only in those patients with truly severe TBI (Lee 2015). Patients in the Eurotherm3235 trial, including all TBI patients with ICP greater than 20 mmHg for 5 min after stage 1, might not be the right population to support the value of hypothermia. As a matter of fact, some controversial therapies may be effective only in more critically ill patients. Therefore, it was not surprising to observe that some patients did well no matter what therapy was used. The Eurotherm3235 trial did not compare the combination of TH and standard therapy to standard therapy alone. In the TH group, mannitol and hypertonic saline were not given unless hypothermia failed to control ICP, which differed from the practice of many other medical centers where TH was used synchronously with standard therapy. In the Eurotherm3235 trial, hypothermia alone was compared to the combination of mannitol and hypertonic saline. Additionally, many independent variables that might affect the long-term clinical outcomes, such as nutrition and advanced rehabilitation capabilities, that might affect the outcome rather significantly. Control of these factors was difficult after patients were discharged from the hospital. Additionally, more patients in the NT group of the Eurotherm3235 trial, though there was no statistical difference, had decompressive craniectomies, which might decrease intracranial hypertension more effectively and influence the outcomes between the TH and NT groups.

Several limitations were present in our meta-analysis. First, the majority of involved RCTs were single-center studies that were assessed to have a high risk of bias, which might confound the effects of TH. Additional high quality and better-designed multi-center studies are needed in the future. Second, the starting time and duration of TH and the protocol of rewarming were different among the involved studies, which increased the risk of bias. Third, significant heterogeneities were detected among the studies involved in the present meta-analysis when we analysed unfavorable clinical outcomes at 3 and 6 months post-TBI, which might confound the results, as heterogeneity was one of the major concerns in the meta-analysis for validity.

## Conclusions

Our meta-analysis demonstrated that therapeutic hypothermia failed to decrease mortality and unfavorable clinical outcomes at 3 months post-TBI or 6 months post-TBI, and might increase the risk of developing pneumonia and cardiovascular complications.

## Abbreviations

TH: therapeutic hypothermia
NT: normothermia treatment
RCT: randomized controlled trial
TBI: traumatic brain injury
TTM: target temperature management
BTF: the Brain Trauma Foundation
